# Prevalence and determinants of diabetes among older adults in Ghana

**DOI:** 10.1186/s12889-016-3845-8

**Published:** 2016-11-21

**Authors:** Samwel Maina Gatimu, Benson Williesham Milimo, Miguel San Sebastian

**Affiliations:** 1 Department of Midwifery and Gender, School of Nursing, Moi University, P.O. Box 4606, 30100 Eldoret, Kenya; 2 Department of Public Health and Clinical Medicine, Umeå International School of Public Health, Umeå University, SE-90 185 Umeå, Sweden; 3 Department of Nursing I, University of the Basque country, Bilbao, Spain; 4 School of Nursing, Moi University, PO. Box 4606, 30100 Eldoret, Kenya

**Keywords:** Diabetes, Prevalence, Ghana, Africa, Determinants, SAGE, Older, Obesity, Ageing

## Abstract

**Background:**

Diabetes is one of the leading non-communicable diseases in Africa, contributing to the increasing disease burden among the old adults. Thus, the aim of this study was to determine the prevalence and determinants of diabetes among adults aged 50 years and above in Ghana.

**Methods:**

A cross sectional study based on data collected from Study of Ageing and Adult Health (SAGE) Wave 1 from 2007 to 2008. Data was collected from 5565 respondents of whom 4135 were aged 50+ years identified using a multistage stratified clusters design. Bivariate and hierarchical multivariable logistic regression models were used to examine the association of the determinants and diabetes.

**Results:**

The weighted prevalence of diabetes among the adults aged 50 years and above in Ghana was 3.95% (95% Confidence Interval: 3.35–4.55) with the prevalence being insignificantly higher in females than males (2.16%, 95% CI: 1.69–2.76 vs. 1.73%, 95% CI: 1.28–2.33). Low level of physical activity (Adjusted Odds Ratio [AOR] 2.11, 95% CI: 1.21–3.69) and obesity (AOR 4.81, 95% CI: 1.92–12.0) were associated with increased odds of diabetes among women while old age (AOR 2.58, 95% CI: 1.29–5.18) and university (AOR 12.8, 95% CI: 4.20–39.1), secondary (AOR 3.61, 95% CI: 1.38–9.47) and primary education (AOR 2.71, 95% CI: 1.02–7.19) were associated with increased the odds of diabetes among men.

**Conclusion:**

The prevalence of diabetes among old adults shows a similar trend with that of the general population. However, the prevalence may have been underestimated due to self-reporting and a high rate of undiagnosed diabetes. In addition, the determinants of diabetes among older adults are a clear indication of the need for diabetes prevention programme targeting the young people and that are gender specific to reduce the burden of diabetes at old age. Physical activity and nutrition should be emphasised in any prevention strategy.

## Background

Approximately 1.9% of the global disability adjusted life years is attributed to diabetes having doubled since 1990 [1]. The International Diabetes Federation (IDF) estimates that 450 million people are living with diabetes, with 5.1 million dying from it annually worldwide [2, 3]. The prevalence of diabetes is expected to double by 2030 from 8.3 to 17.6% globally [2, 4, 5], excluding the high numbers of undiagnosed cases estimated at 175 millions [2, 6]. In sub-Saharan Africa, 21.5 million people are living with diabetes leading to approximately half a million diabetes-related deaths in 2013 [2].

The prevalence of diabetes varies in different age groups with the older population being at a higher risk compared to the young population [7]. For instance, the prevalence of diabetes has been estimated to be between 7.7 to 20% and 5 to 8.8% for adults aged 45 years and more in Kenya and South Africa respectively [7, 8]. In addition, more diabetic people live in urban than in rural areas [8, 9].

Cross-country studies have revealed differences in social and behavioural factors of subjective well being and disability among diabetes patients as well as in the role gender plays in the well being of these patients [10, 11]. In this regard, country-specific studies have found association between diabetes and socioeconomic factors such as education, employment status, wealth and social class [12–14]. Diabetes has also been related with behavioural characteristics of the population such as physical inactivity, poor dietary intake, inadequate intake of fruits and vegetables, tobacco use and alcohol consumption [8].

Despite the demographic transition occurring in Africa, few studies have focused on understanding the magnitude of diabetes among older adults in specific countries [12, 15]. Country-specific studies on the social and behavioural determinants of diabetes have been recommended to guide the development of local diabetes prevention measures and policies [10, 11]. Specifically, in Ghana, studies in the general population have estimated that between 3.3 and 6% of the population has diabetes with the prevalence increasing with age and being higher in urban than in rural areas [2, 15–17]. However, there has been little focus on the health status of the increasing old population, which, for instance has increased from 4.9% in 1960 to 12% in 2010 [18]. Thus, in order to provide more evidence about the magnitude of diabetes among the old population, this study assessed the prevalence and the socioeconomic and behavioural risk factors of diabetes among adults aged 50 years and over in Ghana.

## Methods

### Study design

We conducted a cross sectional study based on data collected in an on-going longitudinal Study of Ageing and Adult Health (SAGE) Wave 1 from 2007 to 2008 in Ghana. SAGE Wave 1 used a multistage stratified clusters design to identify the 5269 households and 5573 people sampled [19, 20]. All household members aged 50 plus years in selected households were invited to participate in the study while one person was randomly selected for households with no person aged 50 years and above [21].

A trained data collection team conducted face to face interviews with the respondents [19, 22] using standardized questionnaires translated into three local languages of Akan, Twi and Ga [19]. An individual questionnaire was used to collect the socio-demographic characteristics, work history and benefits, health state descriptions, anthropometrics, performance tests and biomarkers, risk factors and preventive health behaviours. More information regarding SAGE has been published by the WHO SAGE group elsewhere [20–22].

### Measures

#### Socio-demographics variables

The interviewers recorded the respondents sex as either male or female based on their observation while the respondents reported their age in years, which was then categorised into 50–59 years, 60–69 years and 70 plus years’ age brackets. Information on the area of residence (rural/urban) and their marital status (never married, currently married or cohabiting, and separated, divorced or widowed) was also gathered.

#### Socioeconomic variables

Respondents were asked to report their highest level of education as categorised in 4 groups: primary, secondary, college and university. Wealth quintiles were generated based on household assets through principal component analysis with quintile 1 representing the poorest and quintile 5 representing the wealthiest households [21]. Employment was assessed based on 2 questions; “Have you ever worked?” and “Who is/was your employer in your current/most recent main job? [21].

#### Health behaviours

The Global Physical Activity Questionnaire (GPAQ) that measures the intensity, duration, and frequency of physical activity in occupation, transport-related and leisure time was used. The total time spent, number of days and intensity in physical activity during a typical week were used to generate three levels of physical activity – low, moderate and high [23, 24]. Alcohol and smoking was assessed based on WHO STEPwise approach to surveillance on the Non-communicable diseases Risk Factors (STEPS). A 24-hour recall on the intake of fruits and vegetable consumption based on ‘How many servings of fruit do you eat on a typical day?’ and ‘How many servings of vegetables do you eat on a typical day?’ was used [21]. Respondents who consumed less than five servings of both fruits and vegetable in a week were classified to have insufficient intake while those with who consumed five or more servings of both fruits and vegetable in a week were classified to have sufficient intake [21].

#### Anthropometric variables

The respondents’ weight and height were measured using standard equipment. Body Mass Index (BMI) was calculated based on weight in kilogram (kg) and height in squared metres (m^2^). Respondents were then classified based on BMI as underweight (<18.5 kg/m^2^), normal (18.5 to 24.9 kg/m^2^), overweight (25 to 29.9 kg/m^2^) and obese (>30 kg/m^2^).

#### Outcome variable

Self reported diabetes was assessed based on two questions – “Have you ever been diagnosed with diabetes?” and “Have you ever been told by the doctor to have high blood sugar?” while excluding those with diabetes associated with a pregnancy [21].

### Statistical analysis

Descriptive statistics were used to describe the weighted sample characteristics and the prevalence of the diabetes. The association between the prevalence of diabetes and risk factors was performed using the chi square test. The individual and household survey weights which been calculated based on the selection probability at each stage of selection and were post stratified by sex, locality, region and age groups (for individual), and region and locality (for household) as per the 2009 statistics were used in weighting [19, 20].

Bivariate and multivariable logistic regression models were used to examine the association of socioeconomic and health behavioural variables with diabetes. A multivariable logistic regression was adjusted for the socio-demographic, socioeconomic, behavioural and anthropometric factors and stratified by gender. We conducted gender specific hierarchical models to assess the associated effects of the different confounders on the relationship between diabetes and socio-demographic, socioeconomic, behavioural and anthropometric factors. We adjusted for socio-demographic factors (age and residence) in model I, socio-demographic and socioeconomic factors (education and wealth) in model II and socio-demographic, socioeconomic and health behavioural factors (smoking, alcohol and physical activity) in model III. In the final model IV, adjustment was made for all factors in model III plus anthropometric factor (Body Mass Index). All statistical analysis were performed using STATA 13 [25] and odds ratios with *p*-values of less than 0.05 were considered statistically significant.

### Ethics

The WHO Ethical Review Board and the local review board in Ghana approved SAGE Wave 1. Informed consent was sought from all the respondents and confidentiality maintained [21].

## Results

### Descriptive characteristics of the respondents

Table 1 illustrates the demographic, socioeconomic, health-related behavioural and anthropometric characteristics of the respondents. Out of the 4305 respondents aged 50 plus years included in the study, 216 of them with missing data were dropped, remaining with 4089 respondents who were included the final analysis. Fifty-two per cent of the respondents were males (*n* = 2150) while 48% (*n* = 1983) were females. Sixty per cent of them lived in the rural areas, 59.3% were married and 77% were self-employed. The respondents were equally distributed along the five household wealth quintiles with 20.9% being in the highest wealth quintile while 18.3% in the lowest wealth quintile.

**Table 1:** Demographic, socioeconomic and behavioural characteristic of the respondents

Characteristics	Male	Female	Total	*p*-value^a^
	*n* (%)	*n* (%)	%	
Age				
50–59 years	904 (41.0)	707 (38.9)	40.1	
60–69 years	612 (26.5)	528 (28.3)	27.3	0.48
70 years and above	611 (32.5)	727 (32.8)	32.6	
Residence				
Urban	1342 (59.9)	1093 (59.0)	59.5	0.59
Rural	785 (40.1)	869 (41.0)	40.5	
Marital Status				
Never Married	24 (1.3)	24 (1.3)	1.3	
Currently Married/Cohabiting	1785 (85.4)	537 (30.9)	59.3	<0.01
Separated/Divorced/Widowed	318 (13.3)	1401 (67.9)	39.4	
Level of Education				
No Schooling	957 (43.5)	1303 (65.6)	54.1	
Primary or <6 year Schooling	471 (22.5)	379 (20.0)	21.3	<0.01
Secondary/High School	594 (28.9)	241 (12.3)	20.9	
College/University	105 (5.1)	39 (2.1)	3.7	
Wealth Quintile				
1 (Lowest)	404 (16.4)	411 (20.4)	18.3	
2	382 (17.1)	419 (21.2)	19.1	
3	425 (20.2)	400 (21.6)	20.9	<0.01
4	443 (21.7)	396 (19.9)	20.8	
5 (Highest)	471 (24.6)	333 (16.9)	20.9	
Type of Employer				
Never Employed/Work	34 (1.4)	27 (2.0)	1.6	
Public	304 (15.0)	95 (5.1)	10.3	
Private	120 (5.5)	34 (1.8)	3.8	<0.01
Self-employed	1520 (70.9)	1666 (83.6)	77.0	
Informal	149 (7.2)	140 (7.4)	7.3	
Physical Activity				
Low	396 (21.4)	581 (29.2)	25.1	
Moderate	256 (12.1)	234 (12.2)	12.2	<0.01
High	1453 (66.5)	1127 (58.6)	62.7	
Alcohol Consumption				
Non Drinkers	654 (33.1)	1044 (53.4)	42.8	
Daily Drinkers	690 (31.4)	201 (11.5)	21.9	<0.01
Occasional Drinkers	482 (22.6)	421 (22.1)	22.3	
Former Drinkers	269 (12.9)	260 (13.0)	13.0	
Smoking				
Non-Smokers	1222 (59.9)	1802 (91.6)	75.1	
Current Daily Smokers	327 (12.0)	74 (3.9)	8.1	<0.01
Former Smokers	570 (28.1)	85 (4.5)	16.8	
Fruits/Vegetables Intake				
Adequate	405 (21.1)	426 (22.2)	21.6	0.45
Inadequate	1722 (78.9)	1536 (77.8)	78.4	
Body Mass Index				
Underweight	426 (20.0)	359 (18.8)	19.4	
Normal	1180 (55.2)	887 (46.6)	51.1	<0.01
Overweight	299 (15.2)	338 (18.0)	16.5	
Obese	164 (9.6)	310 (16.6)	12.9	

^a^Pearson Chi Square Test (*χ*2) was used to assess the association between the sex and other socio-demographic, socioeconomic, health behaviours and anthropometric factors

Most of the respondents (62.7%) had high level of physical activity while 25.1% had low level of physical activity. However, 16.5 and 12.9% of the respondents were overweight and obese respectively. Approximately, 80% of the respondents consumed less than five servings of both fruits and vegetables in a week, while 21.9% consumed alcohol daily with 8.1% being daily users of tobacco and its related products.

### Prevalence of diabetes

The weighted prevalence of diabetes was 3.95% (95% CI: 3.35–4.55). The prevalence was higher in females compared to males (2.16%, 95% CI: 1.69–2.76 vs. 1.73%, 95% CI: 1.28–2.33) though not statistically significant (*p*-value = 0.07). Table 2 illustrates the prevalence of diabetes according to demographic, socioeconomic, behavioural and anthropometric characteristics. Respondents in urban areas had a significantly high prevalence compared to those in rural areas. In addition, respondents aged 60 to 69 years had a prevalence of 5.62% (95% CI: 3.88–8.08) while those aged 50 to 59 years had a prevalence of 2.83% (95% CI: 1.99–4.01). A significant association between diabetes and highest level of education was also observed with the highest prevalence (15.2%, 95% CI: 8.70–22.7) among respondents with college and university education and the lowest prevalence (2.6%, 95% CI: 1.92–3.53) among those with no formal schooling. Moreover, high wealth households had significantly higher prevalence compared to low wealth households (7.2%, 95% CI: 5.33–9.45) vs. 2.24%, 95% CI: 1.34–3.79). Furthermore, respondents with low level of physical activity had a significantly higher prevalence of diabetes (6.74%, 95% CI: 0.42–8.51) compared to those with high level of physical activity (2.32%, 95% CI: 0.42–3.15). Respondents who were obese had a prevalence of 9.1% (95% CI: 6.48–13.2) while those with normal BMI had a prevalence of 1.52% (95% CI: 0.81–2.89).

**Table 2:** Prevalence of diabetes according to socioeconomic and health behavioural characteristics of the respondents

Characteristics	Male	Female	Total	*P*-value (*χ*2)^a^
	% (95% CI) [*n* = 73]	% (95% CI) [*n* = 89]	% (95% CI) [*n* = 162]	
Age				
50–59 years	2.44 (1.52–3.95)	3.25 (2.08–5.08)	2.83 (1.99–4.01)	
60–69 years	5.75 (3.50–8.66)	5.75 (3.55–9.05)	5.62 (3.88–8.08)	0.01
70 years and above	2.55 (1.45–4.59)	4.90 (3.51–7.01)	3.74 (2.70–5.17)	
Residence				
Rural	1.94 (1.21–3.15)	2.71 (1.79–4.18)	2.33 (1.65–3.27)	
Urban	5.46 (3.61–7.93)	7.06 (5.35–9.25)	6.19 (4.75–8.03)	<0.01
Level of Education				
No Schooling	1.04 (0.53–2.11)	3.72 (2.71–5.09)	2.60 (1.92–3.53)	
Primary	3.01 (1.61–5.59)	4.42 (2.75–7.07)	3.65 (2.50–5.32)	<0.01
Secondary/High School	4.76 (3.01–6.82)	8.12 (4.73–15.8)	5.71 (4.02–7.68)	
College/University	16.5 (9.50–27.1)	8.18 (2.63–23.4)	15.2 (8.70–22.7)	
Wealth Quintile				
1 (Lowest)	0.61 (0.19–1.97)	3.66 (2.10–6.41)	2.24 (1.34–3.79)	
2	2.27 (1.10–4.78)	1.70 (0.84–3.50)	1.97 (1.20–3.30)	
3	1.88 (1.00–3.53)	3.11 (1.70–5.72)	2.49 (1.59–3.92)	<0.01
4	3.73 (2.14–6.53)	7.03 (4.24–11.2)	5.24 (3.58–7.54)	
5 (Highest)	6.81 (4.29–10.1)	7.82 (5.57–11.0)	7.20 (5.33–9.45)	
Type of Employer				
Never Employed	0.00 (0.00–0.00)	4.42 (1.11–21.5)	2.53 (0.66–11.4)	
Public	6.75 (3.63–10.8)	6.48 (3.12–13.0)	6.69 (4.06–9.85)	
Private	6.90 (3.37–14.0)	0.00 (0.00–0.00)	5.28 (2.64–10.9)	0.02
Self-employed	2.65 (1.81–3.89)	4.65 (3.56–5.96)	3.69 (2.89–4.68)	
Informal	1.09 (0.29–4.70)	2.53 (0.99–6.85)	1.79 (0.85–4.20)	
Physical Activity				
Low	5.93 (3.55–8.90)	7.38 (5.63–9.62)	6.74 (0.42–8.51)	
Moderate	5.21 (3.00–9.07)	7.05 (4.28–12.1)	6.10 (0.42–9.01)	<0.01
High	2.18 (1.42–3.41)	2.49 (1.64–3.80)	2.32 (0.42–3.15)	
Fruits/Vegetables Intake				
Adequate	3.27 (1.63–5.26)	4.78 (2.81–7.59)	4.01 (2.61–5.46)	
Inadequate	3.37 (2.44–4.75)	4.42 (3.47–5.74)	3.87 (3.11–4.92)	0.85
Alcohol				
Non Drinkers	2.18 (1.26–3.95)	4.34 (3.27–5.84)	3.47 (2.70–4.58)	
Daily Drinkers	2.29 (1.21–4.35)	0.67 (0.17–2.68)	1.88 (1.01–3.55)	<0.01
Occasional Drinkers	5.23 (2.79–8.53)	4.74 (2.85–7.44)	4.99 (3.26–6.97)	
Former Drinkers	5.61 (3.11–9.96)	7.22 (3.79–13.4)	6.38 (4.02–10.1)	
Smoking				
Non-Smokers	3.29 (2.32–4.78)	4.65 (3.70–5.95)	4.09 (3.36–5.10)	
Daily Smokers	1.84 (0.54–6.10)	4.01 (1.07–14.6)	2.34 (0.96–5.68)	0.41
Former Smokers	4.17 (2.37–6.43)	1.79 (0.13–6.29)	3.86 (2.16–5.74)	
Body Mass Index				
Underweight	1.09 (0.38–3.05)	2.02 (0.91–4.59)	3.11 (2.33–4.22)	
Normal	3.08 (2.08–4.61)	3.16 (2.08–4.87)	1.52 (0.81–2.89)	<0.01
Overweight	4.35 (2.22–6.99)	4.60 (2.71–7.71)	4.48 (2.92–6.29)	
Obesity	7.74 (4.22–14.5)	9.97 (6.91–14.6)	9.10 (6.48–13.2)	

The values are presented in percentages with the 95% confidence interval in the bracket

^a^Pearson Chi Square Test (*χ*2) was used to assess the relationship between the prevalence of diabetes and the socio-demographic, socioeconomic, health behaviours and anthropometric factors

### Risk factors of diabetes among older adults

Table 3 illustrates the findings of the bivariate and multivariable logistic regression. Respondents aged 60 to 69 years were twice likely to have diabetes compared to those aged 50 to 59 years respectively. In addition, respondents with university and secondary education were five and two times more likely to have diabetes than those who had never been to school respectively. Moreover, respondents with low and moderate level of physical activity were 67 and 117% respectively more likely to be diabetic compared to those with high level of physical activity while those who were obese were two times more likely to be diabetes compared to those of normal BMI.

**Table 3:** Multivariable regression analyses of the socioeconomic determinants and health risk behaviours for both males and females

Characteristics	Univariate	Model 1	Model 2	Model 3	Model 4
	OR (95% CI)	AOR (95% CI)	AOR (95% CI)	AOR (95% CI)	AOR (95% CI)
Sex					
Male	1	1	1	1	1
Female	1.38 (0.96–1.96)	1.34 (0.93–1.93)	1.81 (1.23–2.67)*	1.63 (1.07–2.50)*	1.50 (0.98–2.30)
Age					
50–59 years	1	1	1	1	1
60–69 years	2.04 (1.21–3.45)	2.10 (1.25–3.55)*	2.41 (1.41–4.14)*	2.32 (1.32–4.07)*	2.34 (1.28–4.28)*
70 years and above	1.33 (0.80–2.23)	1.39 (0.83–2.35)	1.96 (1.14–3.37)*	1.70 (0.96–3.01)	1.91 (1.05–3.44)*
Residence					
Rural	1	1	1	1	1
Urban	2.77 (1.77–4.34)	2.81 (1.80–4.38)*	1.87 (1.14–3.07)*	1.45 (0.85–2.45)	1.27 (0.75–2.15)
Education Level					
No schooling	1		1	1	1
Primary School	1.42 (0.88–2.27)		1.48 (0.89–2.47)	1.44 (0.85–2.44)	1.30 (0.76–2.19)
Secondary/High	2.21 (1.41–3.46)		2.28 (1.33–3.89)*	2.21 (1.29–3.79)*	2.08 (1.19–3.65)*
University	6.24 (3.30–11.8)		5.14 (2.44–10.8)*	4.90 (2.30–10.5)*	5.07 (2.35–10.9)*
Wealth					
1 (Lowest)	1		1	1	1
2	0.88 (0.45–1.71)		0.81 (0.42–1.56)	0.71 (0.36–1.38)	0.64 (0.32–1.27)
3	1.11 (0.56–2.21)		0.92 (0.48–1.78)	0.84 (0.43–1.66)	0.80 (0.40–1.59)
4	2.38 (1.23–4.58)		1.58 (0.80–3.12)	1.49 (0.72–3.07)	1.18 (0.58–2.41)
5 (Highest)	3.31 (1.81–6.08)		1.61 (0.81–3.17)	1.46 (0.72–2.96)	1.14 (0.54–2.39)
Physical Activity					
High	1			1	1
Moderate	2.77 (1.77–4.34)			2.15 (1.32–3.50)*	2.17 (1.32–3.56)*
Low	2.96 (2.06–4.23)			1.88 (1.24–2.85)*	1.67 (1.12–2.50)*
Alcohol					
Non Drinkers	1			1	1
Daily Drinkers	0.53 (0.27–1.06)			0.65 (0.33–1.28)	0.59 (0.29–1.20)
Occasional Drinkers	1.37 (0.87–2.18)			1.30 (0.81–2.10)	1.27 (0.77–2.09)
Former Drinkers	1.87 (1.09–3.21)			1.54 (0.89–2.68)	1.57 (0.90–2.76)
Fruits/Vegetables Intake					
Adequate	1			1	1
Inadequate	1.03 (0.68–1.58)			1.12 (0.72–1.72)	1.17 (0.69–2.90)
Smoking					
Never Smoker	1			1	1
Daily/Not Daily	0.56 (0.22–1.43)			1.39 (0.52–3.72)	1.08 (0.38–3.08)
Not Current Smoker	0.85 (0.52–1.38)			1.07 (0.64–1.80)	1.19 (0.71–2.01)
Body Mass Index					
Normal	1				1
Underweight	2.08 (1.07–4.05)				1.85 (0.97–3.55)
Overweight	2.88 (1.41–5.92)				1.91 (0.93–3.92)
Obese	6.57 (3.03–14.3)				3.61 (1.69–7.70)*

Model I: Adjusted for socio-demographic factors (age and residence)

Model II: Adjusted for socio-demographic factors (age and residence) and socioeconomic factors (education and wealth)

Model III: Model II plus further adjustment for health behaviours (smoking, alcohol use, fruits and vegetable intake and physical activity)

Model IV: Model III with additional adjustment for anthropometric factor (Body mass index)

*Significance level (*p* < 0.05), *AOR* adjusted odd ratio, *CI* Confidence Interval

However, when stratifying by sex, men aged 60 to 69 years (AOR 2.58, 95% CI: 1.29–5.18) and those with primary (2.71, 95% CI: 1.02–7.19), secondary (3.61, 95% CI: 1.38–9.47) and university (12.8, 95% CI: 4.20–39.1) education showed an associated increase in risk of diabetes (Table 4). On the other hand, women who were obese (4.81, 95% CI: 1.92–12.0) and with low level of physical activity (2.11, 95% CI: 1.21–3.69) had an associated increase in the odds of diabetes while those who consume alcohol daily (0.18, 95% CI: 0.04–0.82) and in the second wealth quintile (0.33, 95% CI: 0.12–0.91) had an associated reduction in odds of diabetes (Table 5).

**Table 4:** Multivariable regression analyses of the socioeconomic determinants and health risk behaviours for males

Characteristics	Model 1	Model 2	Model 3	Model 4
	AOR (95% CI)	AOR (95% CI)	AOR (95% CI)	AOR (95% CI)
Age				
50–59 years	1	1	1	1
60–69 years	2.45 (1.26–4.72)*	2.70 (1.42–5.13)*	2.77 (1.37–5.59)*	2.58 (1.29–5.18)*
70 years and above	1.11 (0.51–2.43)	1.61 (0.91–3.62)	1.63 (0.74–3.62)	1.78 (0.80–3.97)
Residence				
Rural	1	1	1	1
Urban	2.91 (1.54–5.53)*	1.81 (0.91–3.62)	1.54 (0.75–3.16)	1.60 (0.76–3.38)
Education Level				
No schooling		1	1	1
Primary School/Less		2.83 (1.12–7.15)*	3.41 (1.29–9.04)*	2.71 (1.02–7.19)*
Secondary/High		3.71 (1.60–8.61)*	3.70 (1.46–9.40)*	3.61 (1.38–9.47)*
University		12.2 (4.47–33.3)*	12.3 (4.14–36.6)*	12.8 (4.20–39.1)*
Wealth				
1 (Lowest)		1	1	1
2		3.22 (0.84–12.4)	2.63 (0.84–10.5)	2.44 (0.63–9.43)
3		1.98 (0.53–7.32)	2.23 (0.53–8.81)	1.84 (0.49–6.89)
4		3.13 (0.76–13.0)	3.29 (0.76–15.0)	1.97 (0.48–8.15)
5 (Highest)		3.93 (1.01–15.4)*	4.29 (0.99–18.6)	2.60 (0.65–10.5)
Physical Activity				
High			1	1
Moderate			1.78 (0.83–3. 81)	1.58 (0.71–3.56)
Low			1.70 (0.83–3.48)	1.32 (0.67–2.59)
Alcohol				
Non Drinkers			1	1
Daily Drinkers			1.02 (0.46–2.27)	0.90 (0.41–2.01)
Occasional Drinkers			1.80 (0.80–4.06)	1.79 (0.78–4.09)
Former Drinkers			1.91 (0.80–4.52)	1.91 (0.82–4.49)
Fruits/Vegetables Intake				
Adequate			1	1
Inadequate			1.41 (0.70–2.86)	1.42 (0.69–2.90)
Smoking				
Never Smoker			1	1
Daily/Not Daily			1.63 (0.44–6.03)	0.76 (0.20–2.86)
Not Current Smoker			1.28 (0.71–2.30)	1.35 (0.76–2.39)
Body Mass Index				
Normal				1
Underweight				2.41 (0.81–7.22)
Overweight				2.48 (0.73–8.40)
Obese				3.74 (0.93–15.1)

Model I: Adjusted for socio-demographic factors (age and residence)

Model II: Adjusted for socio-demographic factors (age and residence) and socioeconomic factors (education and wealth)

Model III: Model II plus further adjustment for health behaviours (smoking, alcohol use, fruits and vegetable intake and physical activity)

Model IV: Model III with additional adjustment for anthropometric factor (Body mass index)

*Significance level (*p* < 0.05), *AOR* adjusted odd ratio, *CI* confidence Interval

**Table 5:** Multivariable regression analyses of the socioeconomic determinants and health risk behaviours for females

Characteristics	Model 1	Model 2	Model 3	Model 4
	AOR (95% CI)	AOR (95% CI)	AOR (95% CI)	AOR (95% CI)
Age				
50–59 years	1	1	1	1
60–69 years	1.84 (0.93–3.66)	2.08 (1.01–4.31)*	1.75 (0.85–3.62)	1.81 (0.85–3.85)
70 years and above	1.63 (0.86–3.07)	2.09 (1.07–4.08)*	1.56 (0.75–3.22)	1.83 (0.88–3.80)
Residence				
Rural	1	1	1	1
Urban	2.74 (1.62–4.63)*	1.95 (1.03–3.70)*	1.47 (0.77–2.80)	1.07 (0.56–2.05)
Education Level				
No schooling		1	1	1
Primary School /Less		1.11 (0.56–2.19)	0.96 (0.46–1.97)	0.90 (0.45–1.82)
Secondary /High		1.89 (0.81–4.41)	1.84 (0.79–4.26)	1.55 (0.63–3.81)
University		1.48 (0.37–5.97)	1.34 (0.33–5.48)	1.23 (0.32–4.70)
Wealth				
1 (Lowest)		1	1	1
2		0.44 (0.17–1.12)	0.39 (0.15–1.01)	0.33 (0.12–0.91)*
3		0.76 (0.35–1.66)	0.57 (0.25–1.28)	0.55 (0.23–1.29)
4		1.44 (0.65–3.17)	1.21 (0.52–2.81)	1.07 (0.43–2.67)
5 (Highest)		1.34 (0.58–3.10)	1.02 (0.44–2.39)	0.87 (0.33–2.30)
Physical Activity				
High			1	1
Moderate			2.79 (1.34–5.78)*	3.20 (1.53–6.69)*
Low			2.17 (1.25–3.76)*	2.11 (1.21–3.69)*
Alcohol				
Non Drinkers			1	1
Daily Drinkers			0.18 (0.04–0.81)*	0.18 (0.04–0.82)*
Occasional Drinkers			1.05 (0.58–1.88)	0.97 (0.52–1.83)
Former Drinkers			1.55 (0.75–3.20)	1.68 (0.78–3.61)
Fruits/Vegetables Intake				
Adequate			1	1
Inadequate			0.95 (0.55–1.65)	0.98 (0.54–1.79)
Smoking				
Never Smoker			1	1
Daily/Not Daily			1.70 (0.37–7.84)	0.76 (0.44–11.0)
Former Smoker			1.00 (1.00–1.00)	1.00 (1.00–1.00)
Body Mass Index				
Normal				1
Underweight				1.77 (0.69–4.51)
Overweight				2.03 (0.74–5.57)
Obese				4.81 (1.92–12.0)*

Model I: Adjusted for socio-demographic factors (age and residence)

Model II: Adjusted for socio-demographic factors (age and residence) and socioeconomic factors (education and wealth)

Model III: Model II plus further adjustment for health behaviours (smoking, alcohol use, fruits and vegetable intake and physical activity)

Model IV: Model III with additional adjustment for anthropometric factor (Body mass index)

*Significance level (*p* < 0.05),*AOR* adjusted odd ratio, *CI* confidence Interval

## Discussion

This study sought to assess the prevalence of diabetes among the older adults aged 50 plus years and the demographic, socioeconomic, health behaviour and anthropometric risk factors of diabetes in older adults in Ghana. The overall prevalence was 3.95%, with no statistically significant differences by sex. Old age and education were associated with higher risk of diabetes among men while low and moderate levels of physical activity and obesity were associated with higher risk of diabetes among women.

The prevalence of diabetes among old adults in Ghana was within the range of the prevalence of diabetes in the general population of Ghana of between 3.8 and 6.3% [15, 17]. However, the prevalence rate was lower in comparison to Nigeria, South Africa and Kenya, which could be due to differences in population size, exposures to risk factors and study designs [26–29].

Similar to our study, most studies in Ghana and Africa have found no statistical difference in diabetes between men and women [7–9, 12, 15, 26]. However, our findings differ from a study by Amoah and colleagues (2002) in urban Accra that found that males had a statistically higher prevalence than females, which could partly be explained by the high population of older males in the study [17].

The prevalence of diabetes among older adults increased with age from 1.13 to 1.57% among 50 to 59 years and 60 to 69 years respectively. This is a pattern observed in other studies in Ghana, which could reflect the ageing population in Ghana, currently constituting 12% of the population [15–17, 23]. Specifically, old age was found to be associated with increased risk of diabetes only among males. This association could be explained by the cumulative effect of early life exposure to biological, social and behavioural determinants of diabetes [30].

We also found that the prevalence of diabetes significantly increased with advancing levels of education. It was high among those with university and college education compared to those in other levels of education, both in the general population and among males. There exist inequalities in education favouring males over females in Ghana [31] which, was also noted in our study where males had significantly higher level of education compared with females. High education has also been linked to the increasing adoption of new sedentary lifestyles, changes in dietary intake and less engagement in physical activity due to the nature of job and pressure at work thus increasing their cumulative health risk [32–35]. This is further supported in our study where education among male was significantly associated with poor health behaviours (low level of physical inactivity, smoking and alcohol) and socioeconomic factors (urban residence and wealth).

Obesity has been identified as independent risk factor to diabetes in previous studies from Ghana [15, 16]. This was also supported by our study where obesity was associated with more than threefold increase in the odds of being diabetic in the general population and fivefold among women. Some studies have also found obesity among women as a key risk factor for diabetes in Africa and have attributed it to a perception in Ghana and Africa in general of large body size being viewed positively and healthy [14, 28, 36, 37]. Moreover, obesity was significantly higher in women than in men in our study. Also, the prevalence of diabetes was significantly higher among obese women compared to obese men. Females have been known to have high rate of biological risk factors such as insulin resistance, abdominal adiposity and low high-density lipoproteins hence an associated increase in the risk of diabetes [38]. The increasing level of obesity in Africa has been attributed to the changing demographic dynamics, urbanization, poverty, nutrition transition and changing lifestyles [37, 39, 40]. Obesity is also associated with increase in body’s energy requirement hence the associated increase in blood glucose and saturated fatty acids and possibly insulin resistance [41].

Physical inactivity was another significant risk factor increasing the odds of diabetes among older adults. In particular, physical inactivity was an independent risk factor of diabetes among women. This is supported by the significantly higher number of women with low level of physical activity compared to male. It is also further explained by studies that have shown physically inactive people to have higher prevalence of diabetes in Ghana [17] with similar observations being made in Kenya and Nigeria [7, 26]. Literature also shows physical inactivity is associated with increase in the risk of obesity by increasing the amount of saturated fatty acids in the body and hence triggering insulin insensitivity [41].

An interesting finding in our study was the associated protective effect of daily consumption of alcohol and low socioeconomic position among women. Studies have found women in low socioeconomic position to be less educated and to have poor access to nutrition [42]. Thus, they may be protected from the sedentary lifestyle that is adopted by those of high socioeconomic position. However, the trend is changing and a number of studies are showing an increasing dual burden of malnutrition in low and middle-income countries [14, 37, 39, 43, 44].

This study identifies some of the major factors associated with diabetes among the older adults in Ghana. The gender difference in the risk factors also confirms the assertion that gender plays a different role among patients with diabetes. In Ghana, female gender has been found to be associated with poor subjective health among patients with diabetes [10, 11]. To reduce the burden of diabetes, health policies targeting specific risk factors of diabetes in Ghana ought to be formulated. In addition, specific measures should be taken to address the increasing rate of obesity and physical inactivity as well as address the social determinants of diabetes such as education.

### Strengths and limitations of the study

One of the strengths of the study is the use of a large sample size that is representative of the whole country and can thus be used for comparison. The study also had a high response rate of 86% with few missing data hence making the results more reliable. However, being of cross sectional study, we are limited in assessing causation or direction of association and studying prevalence over time. Our study may also have underestimated the prevalence of diabetes because of under-reporting as a result of self-reporting of diabetes, which does not account for the high rate of undiagnosed cases of diabetes estimated at 62% in Africa [2]. Moreover, laboratory data such as fasting plasma glucose and HbA1c are needed to correctly estimate the prevalence of diabetes [45]. In addition, alcohol and smoking were also measured based on whether the respondents had ever been exposed providing a crude measure that may not reflect the real exposures. Furthermore, type 1 and type 2 diabetes have not been differentiated despite the varying prevalence and risk factors among old adults. Lastly, some of the known risk factors of diabetes such as genetic predisposition were not investigated in this study.

## Conclusions

In conclusion, our study provides evidence on the prevalence of diabetes in Ghana and its associated risk factors among older adults. The prevalence of diabetes among old adults in Ghana was 3.95%. Old age, higher education, low level of physical activity and obesity were associated with higher risk of diabetes in the general population. Gender differences exist in the risk factors of diabetes with old age and higher education being the risk factors among males and obesity and physical inactivity as risk factors among females. Hence, there is need to enhance the existing prevention programmes with emphasis on social determinants of diabetes, sustained physical activities and good nutritional practices starting at an early age to reduce the level of exposure and subsequently to decrease the prevalence of diabetes at old age.

## Abbreviations

AOR: Adjusted odds ratio
BMI: Body mass index
CI: Confidence interval
GPAQ: Global physical activity questionnaire
IDF: International diabetes federation
OR: Odds ratio
SAGE: Study of global AGEing and adult health
STEPS: STEPwise approach to surveillance on the Non-communicable diseases Risk Factors
WHO: World Health Organization
